# Predicting Regional Variations in Nationalism With Online Expression of Disgust in China

**DOI:** 10.3389/fpsyg.2021.564386

**Published:** 2021-05-28

**Authors:** Shuqing Gao, Hao Chen, Kaisheng Lai, Weining Qian

**Affiliations:** ^1^Faculty of Psychology, Beijing Normal University, Beijing, China; ^2^Department of Social Psychology, Nankai University, Tianjin, China; ^3^Research Centre for Greater Bay Area Social Psychology, Sun Yat-sen University, Guangzhou, China; ^4^College of Journalism and Communication, Jinan University, Guangzhou, China; ^5^School of Data Science and Engineering, East China Normal University, Shanghai, China

**Keywords:** disgust, nationalism, social media, Weibo, China

## Abstract

Disgust is one of the basic emotions and is part of the behavioral immune system, which evolutionarily protects humans from toxic substances as well as from contamination threats by outgroup members. Previous works reveal that disgust not only activates humans’ defense against potential individual and collective threats, but also leads to severe moral judgments, negative intergroup attitudes, and even conservative political orientations. As is already known, nationalism is an ideology that features both negative feelings toward outgroups and beliefs about native superiority or privileges. Evidence from previous studies suggests that disgust is related to nationalism’s several components but lacks direct research on nationalism and disgust. The current study examines the relationship between disgust and nationalism in China at both individual and regional levels. In study 1, participants temporally induced disgust (vs. control) increasing the adoption of nationalism. In Study 2, we analyzed covariation in disgust expression in the Chinese micro-blog *Weibo* and the nationalism index as part of an online large-scale political survey http://zuobiao.me/ at the province level across Mainland China. The results show that online expression of disgust positively predicts nationalistic orientation at the regional level. Finally, we discuss how the findings shed light on research concerning online emotion expression and potential future directions.

## Introduction

In recent years, nationalist movements have surged into prominence as demonstrated by the United Kingdom’s Brexit and Donald Trump’s victory in the United States (Gusterson, 2017). Nationalism is becoming a hot issue and has an essential influence on national development and international relations. Previous studies show that disgust causes more negative attitudes toward outgroups and more positive attitudes toward the ingroup (Suedfeld and Schaller, 2002; Hodson and Costello, 2007; Hodson et al., 2013). Nationalism can be regarded as a conservative and negative intergroup value (Kosterman and Feshbach, 1989; Freeden, 1998). Many observers have discovered the rise of global nationalism in 2020 and believe it is related to the global pandemic of COVID-19 (Bieber, 2020; Goode et al., 2020).

Disgust is a particular emotion easily elicited by revolting smells, sights, or words (Inbar et al., 2009b). Most researchers regard disgust as a basic emotion that developed in the process of human evolution and believe that each of the basic emotions has its unique evolutionary function to deal with a specific kind of selection stress (Ekman et al., 1987; Rozin and Fallon, 1987). The function of disgust is related to pathogens. It serves as a disease-avoidance mechanism that makes people avoid contaminated food, dangerous environments, and persons who may carry pathogens (Rozin et al., 2008; Tybur et al., 2009). Hence, disgust elicitors and infectious disease threats can cause individuals to produce specific disgust responses, which basically represent rejection.

Although originating as an adaptive avoidance response, disgust has become more than a mere rejection of inedible foods and has been culturally enriched and coopted by other self-protection systems (Rozin et al., 2000). Researchers have found that people who are induced disgust by exposure to a disgusting stimulus (e.g., a revolting image or smell) would judge moral violations as more immoral and show more negative feelings toward social groups associated with these behaviors (Schnall et al., 2008; Dasgupta et al., 2009). Individuals who are more easily disgusted (higher in disgust sensitivity) show more economic and political conservatism (Inbar et al., 2012).

In terms of intergroup attitudes, compared with members of the ingroup, outgroups are more likely to carry deadly pathogens, lice, and other parasites. In other words, outgroup members have historically been a source of contamination (Suedfeld and Schaller, 2002). Therefore, negative attitudes or prejudice toward outgroups protect us by avoiding them reflecting an adaptive strategy (Terrizzi et al., 2010; Hodson et al., 2013). Thus far, several studies have examined the relationship between disgust and intergroup attitudes based on the disease-avoidance function of disgust. Researchers have found that chronic and contextually aroused feelings of vulnerability to disease motivate xenophobic attitudes toward foreign people (Faulkner et al., 2004). Navarrete and Fessler (2006) found a positive relationship between disease avoidance and ethnocentrism: higher disgust sensitivity scores indicated stronger ethnocentrism. Terrizzi et al. (2012) considered religious conservatism as an evolutionarily evoked disease-avoidance strategy. The most interesting is that women’s disgust sensitivity became elevated in the first trimester of pregnancy to protect the baby’s and the mother’s safety (Żelaźniewicz and Pawłowski, 2015) while, at the same time, their ethnocentrism also increased (Navarrete et al., 2007).

Nationalism is regarded as the detrimental facet of positive ingroup evaluation, holding the view that one’s country is superior to others and should be dominant (Kosterman and Feshbach, 1989; Mummendey et al., 2001). Nationalism is closely linked with ethnocentrism, but both also have differences. Nationalism’s outgroup rejection or negativity is mainly reflected in national groups rather than ethnic groups (Mummendey et al., 2011; Bizumic and Duckitt, 2012; Rothschild, 2015). Apart from this, nationalism is usually seen as an ideology with more abundant meaning than ethnocentrism (Freeden, 1998). Researchers regard ethnocentrism as a critical contributor to nationalism (Bizumic and Duckitt, 2012) and have found that ethnocentrism positively predicts nationalism (Angraini et al., 2014). Considering the previous research findings that disgust can predict ethnocentrism, we think there is a connection between disgust and nationalism.

Nationalism also emphasizes the need for national purity, national power, and even national self-determination compared with ethnocentrism (Angraini et al., 2014). Disgust is also associated with “national purity” and power-seeking. Some studies have found that disgust leads to the pursuit of religious and cultural purity, which helps to prevent people from being contaminated by outgroup pathogens (Terrizzi et al., 2012; Cheon et al., 2016). Additionally, disgust usually increases the accessibility of death-related thoughts (Belmi and Pfeffer, 2016). According to the terror management theory, death-related thoughts increased individuals’ motivation to acquire the power to protect their psychological security (Cox et al., 2007). According to the research mentioned above, we offer our first hypothesis:

***H1. Disgust can predict nationalism at an individual level.***

Like many psychological variables, nationalism has individual differences and variations at the regional macro-level (Lan and Li, 2015). Analyzing the relationship between nationalism and disgust at the regional level can better illustrate the closeness and robustness of their relationship.

Advances in social media technology make it possible to collect and analyze data relevant to the macro-level. Data from increasingly popular social media websites provide valuable opportunities for researchers to study human activities and social issues from both individual and macro perspectives (e.g., Kosinski et al., 2015). People on famous social media websites, such as Facebook, Twitter, and Sina Weibo, occasionally broadcast brief messages to the public. Regardless of the message content or topic, this personal information usually conveys the author’s mood status, representing a microscopic component of the public mood state. Online text sentiment analysis techniques provide technical support and allow the mining and analysis of people’s opinions, attitudes, and emotions based on massive amounts of online information (Nielsen, 2011; Saif et al., 2012; Mitchell et al., 2013). With the tools of sentiment analysis techniques, a few studies have recently indicated that, like individual emotions, macro-level public emotions can predict people’s cognitions, thoughts, and behavior. For example, by analyzing the text content of social media websites, researchers have found that macro-level online emotions could predict county-level heart disease mortality and the national stock market over time (Bollen et al., 2011; Lai et al., 2014; Eichstaedt et al., 2015).

According to the theory of socioecological psychology, people’s psyche and behavior are shaped by natural and social environment factors (Oishi, 2014). The socioecological and cross-psychological perspectives suggest that people in different regions commonly have different psychological traits and behavioral patterns, and there are often associations between them (Rentfrow et al., 2008). For example, researchers found that state-level differences in personality could predict statewide voting patterns in the United States (Rentfrow et al., 2009). Online disgust expression measured by social media data could capture and reflect the regional differences in disgust sensitivity (proneness to experience disgust easily and intensely, Haidt et al., 1994). Accordingly, we hypothesized:

***H2. Online disgust can predict nationalism at the regional level.***

In terms of disgust and intergroup attitudes, the majority of the samples used in previous studies come from Western, educated, industrialized, rich, and democratic (WEIRD) countries. However, in non-WEIRD cultures, the influence of disgust on group attitudes, especially at the national group level, still needs more research evidence. Furthermore, most previous studies’ results are based on small sample surveys or laboratory studies, lacking ecological validity. The current study used a non-WEIRD sample from China. It adopted a big data approach, using social media data of 1.6 million people and large-scale survey data of 150,000 people to analyze the relationship between basic emotions and nationalism.

## Study 1

This study aimed to explore the causal relationship between disgust and nationalism at the individual level. Previous studies have not provided a direct analysis of the relationship between disgust and nationalism. Therefore, this study used an experimental method to confirm that disgust can predict nationalism.

### Method

#### Participants

A total of 891 college students were recruited from four universities in Henan province, China. Eighty-six participants were excluded due to not adhering to the requirements of the experiment (57 participants wrote fewer than 30 words in the recall task. The sentences written by the other 29 participants were not related to recall tasks or even just some meaningless characters (e.g., “11111.”). Finally, there were 805 participants in the final analysis (528 females and 277 males). Participants were aged between 17 and 25 years (*M*_*age*_ = 19.64, SD = 1.22). Informed consent was obtained from all participants before their participation.

#### Procedure

Participants were randomly assigned to one of the two conditions: 384 people in the disgust-induced condition and 421 people in the neutral condition. The experimental tasks for both conditions were borrowed and adapted from previous studies (Charash and McKay, 2002; Luo et al., 2013). In the disgust-induced condition (the disgust group), participants were asked to recall disgusting scenes and to make sentences with words (no less than 30 words), such as “rotten,” “maggot,” “disgusting,” and “vomit.” In the neutral condition (the non-disgust group), participants were asked to recall their experiences 1 day before and to make sentences with words such as “fresh,” “vegetable,” “food,” and “swallow.” After that, participants in both conditions were required to complete a brief questionnaire that included disgust, nationalism, and demographic information.

#### Measures of Key Variables

##### Disgust

To validate our experimental manipulation, we used an item (“The degree to which you feel disgusted now” on a five-point Likert scale) to measure participants’ disgust. Higher scores indicate greater disgust.

##### Nationalism

Participants’ nationalism was measured with eight items adapted from the Patriotism and Nationalism Scale (Kosterman and Feshbach, 1989). Example items are “In view of Chinese economic superiority, it is only right that we should have a bigger say in the United Nations and other international organizations,” and “The important thing for China foreign aid program is to see to it that China gains a political advantage.” Participants rated items from −2 (“strongly disagree”) to 2 (“strongly agree”). After completion, all participants were debriefed. An overall nationalism score of each participant was computed as the mean of the eight items with higher scores reflecting a higher level of nationalistic orientation (Cronbach’s α = 0.76).

### Results

#### Manipulation Check

We tested the disgust manipulation by examining the difference in self-reported disgust between the disgust and non-disgust groups. Using an independent samples *t*-test, we found significantly higher disgust in the disgust group (*M* = 2.92, SD = 1.19) compared with the non-disgust group (*M* = 2.05, SD = 0.95, *t* = 11.43, *p* < 0.001, Cohen’s *d* = 0.81). The results show that the experimental manipulation was successful.

#### Disgust and Nationalism at the Individual Level

To analyze the relationship between disgust and nationalism, we examined the differences in the two groups’ nationalism scores. An independent samples *t*-test showed that the disgust group’s nationalism scores (*M* = 3.03, SD = 0.59) were significantly higher than the non-disgust group’s nationalism scores (*M* = 2.89, SD = 0.56, *t* = 3.38, *p* < 0.001, Cohen’s *d* = 0.24). This result suggests that disgust can influence nationalism at the individual level.

## Study 2

Study 1 confirms the relationship between individual disgust and nationalism. Regarding the macro-level, the question arises whether an area’s disgust can still predict nationalism. This study tested the ecological validity of the relationship between disgust and nationalism and explored the factors influencing nationalism at the macro-level via social media data.

### Method

#### Data Sources

##### Online disgust data

Online emotion data were culled and collected from *Weibo* (or *Sina Weibo*, originally), the most popular micro-blogging website in China, similar to *Twitter* and *Facebook*. Until September 2014, the number of monthly active users on *Weibo* had reached 167 million (Weibo Data Center, 2014). We collected the posts (tweets) of approximately 1.6 million active users across 31 provinces and autonomous regions in Mainland China, from January 1, 2011, to December 31, 2013, via *Weibo*’s API^1^. We identified the province to which each person in the analysis belonged by the user’s profile information. Then, we obtained each person’s emotion data through a Chinese sentiment analysis tool of micro-blog data. In addition to geotags, some users disclosed their gender and verification status. In this sample, males accounted for 66.17%, and verified users accounted for 23.51%. It needs to be pointed out that these posts are all made open to the public by *Weibo* users voluntarily.

##### Nationalism data

Nationalism data came from the *Chinese Political Compass*^2^, a website investigating people’s political, economic, and cultural values. This website asked visitors to appraise 50 items (e.g., “Wasting food is an individual freedom”) on a four-point Likert scale. The publicly available sample of this survey was 171,830 in 2014. Through an analysis of *IP* addresses, we obtained 153,237 participants’ survey data from 31 provinces and autonomous regions in Mainland China. On average, participants were 25.11 years old, and 35.90% of participants were female and 64.10% male.

#### Measures

##### Measurement of disgust online

We used the Weibo Five Basic Mood Lexicon (*Weibo-5BML*), which includes happiness, sadness, fear, anger, and disgust to perform sentiment analysis. *Weibo-5BML* contains a total of 818 emotional terms (happiness has 306 terms, sadness has 205 terms, fear has 72 terms, disgust has 142 terms, and anger has 93 terms), which have been proven to have good reliability and validity in an analysis of micro-blog data (Dong Y. et al., 2015; Dong et al., 2015a,b; Wu et al., 2018). We analyzed texts in posts using a term-based matching technique (O’Connor et al., 2010), which matches the emotional terms used in each tweet against *Weibo-5BML*, which could capture a variety of quantitative emotional information in Weibo tweets and map them into their respective categories of basic emotions. Then, we computed the score of each term that matched *Weibo-5BML* as the fraction of tweets containing it for each year in 31 provinces of Mainland China. After that, we averaged the quantity of overall words of each year in 31 provinces to extract the adjusted ratio of emotional terms. We obtained the scores of five basic emotions in 31 provinces across three years. We found that the scores of each of five basic emotions in 2011, 2012, and 2013 were highly correlated (0.56 ≤ *Pearson*’s *r*s ≤ 0.89), so we averaged each emotion annually from 2011 to 2013 to obtain five basic emotional indexes of 31 provinces.

**FIGURE 1:**
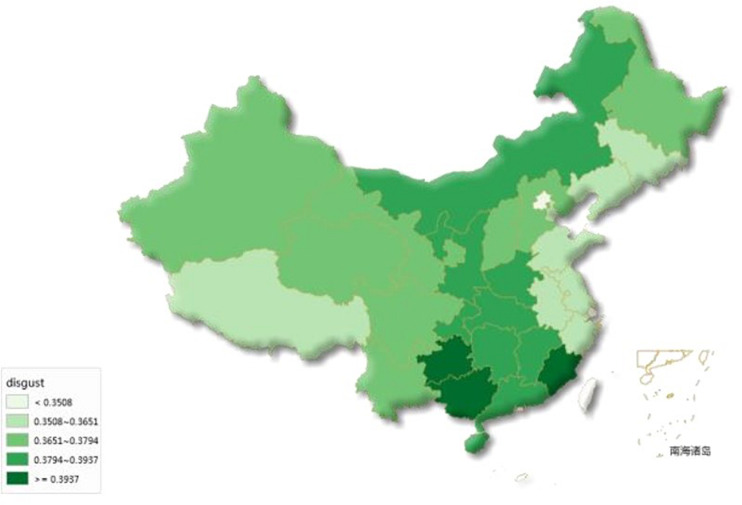
Online disgust of 31 provinces in China, 2011–2013.

Figure 1 shows the distribution of disgust across provinces in China for 2011–2013. Areas with darker colors have greater online disgust expression.

##### Measurement of regional nationalism

**FIGURE 2:**
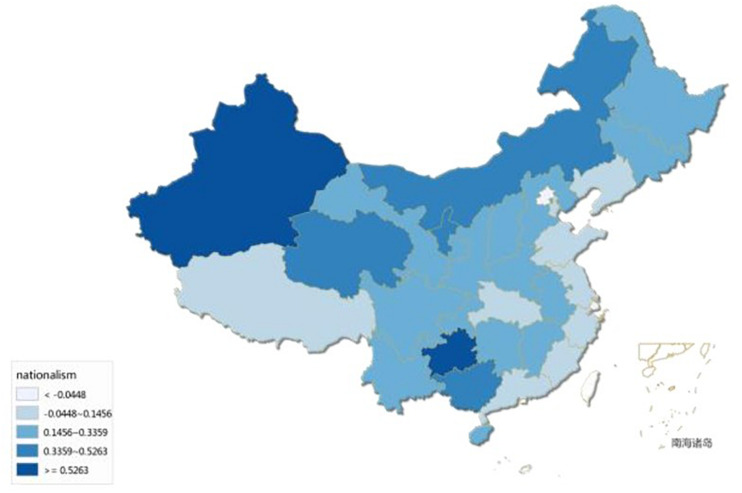
Nationalism of 31 provinces in China, 2014.

Among the 50 items on the *Chinese Political Compass*, seven items measured participants’ nationalism (Wu, 2014). Example items are “The highest interest of society is national territorial integrity,” “If with sufficient comprehensive strength China has the right of any actions to protect its own interest,” and “Human rights have precedence over national sovereignty (reversed score)” (−2 = strongly disagree; 2 = strongly agree). An overall *nationalism score* was computed as the mean of the seven items with higher scores reflecting a higher level of nationalistic orientation (α = 0.69). According to each participant’s IP address, we identified which province the participant was in. We used the average score of all participants in each province as the province’s *nationalism score*. Figure 2 shows the distribution of nationalism across provinces in China in 2014. Areas with darker colors have greater nationalism.

##### Control variables

We also use some regional-level predictors in the ordinary-least-squares regression analysis described below. The control variables include GDP per capita, economic openness, geographical closeness, the share of college students (college students divided by population), and the share of the rural population (rural residents divided by population). Economic openness was computed by the total regional trade (imports plus exports) divided by GDP. The higher the economic openness of an area means the higher the level of globalization in the region. Geographical closeness was the distance from the center of one province (provincial capital city) to the nearest city with a seaport as measured by the electronic map. For example, on an electronic map, the straight-line distance (in 1,000 kilometers) from Beijing (province-level city) to the nearest city (Tianjin Binhai New Area) with a seaport is 129.8, then the geographic closeness of Beijing is 129.8. These control variables were considered to be related to regional nationalism in a previous study (Lan and Li, 2015). The data of these variables in 2014 were obtained from the National Bureau of Statistics of China (NBS;^3^) except for geographical closeness. The data set is available upon request as the authors indicated in the response letter.

### Results

First, we analyzed the relationship between online disgust and nationalistic orientation by correlation and linear regression analysis at the regional level. Then, we examined the relationships between other online basic emotions and nationalism to exclude the alternative emotional explanations of nationalism.

#### Disgust Expression and Nationalism

**FIGURE 3:**
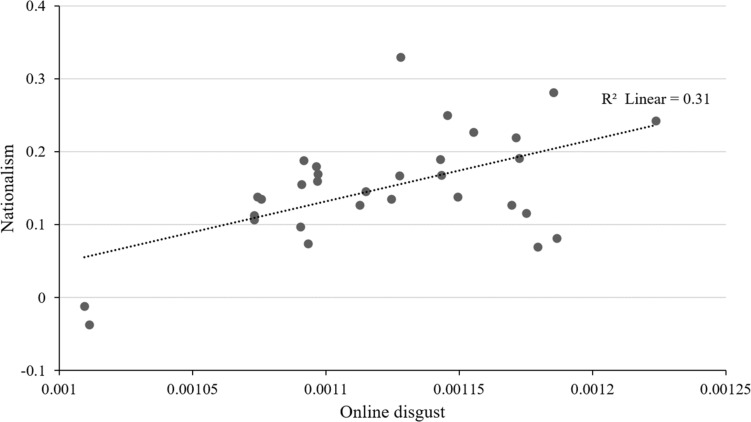
Scatterplot of online disgust and nationalism at the provincial level.

To examine the relationship between online disgust and nationalism, we conducted a correlation analysis. The mean and standard deviation of each variable and correlations of all variables are shown in Table 1. We find a positive correlation between disgust expression and nationalism (*r* = 0.56, *p* = 0.0011) (Figure 3). The preliminary findings confirm the relationship between online disgust and nationalism at the regional level.

**TABLE 1:** Descriptive statistics and correlations among variables.

**Variables**	**1**	**2**	**3**	**4**	**5**	**6**	**7**	**8**	**9**	**10**	**11**
1 Nationalism	–										
2 Online disgust^†^	0.56**	–									
3 Online anger^†^	0.60**	0.45*	–								
4 Online happiness^†^	0.29	0.56**	0.02	–							
5 Online fear^†^	0.51**	0.17	0.69**	−0.15	–						
6 Online sadness^†^	0.61***	0.53**	0.60**	0.55**	0.41*	–					
7 GDP per capita	−0.72***	−0.58**	−0.52**	−0.47**	−0.53**	−0.72***	–				
8 Economic openness	−0.76***	−0.48**	−0.72**	−0.54**	−0.27	−0.71***	0.80***	–			
9 Geographical closeness	0.50**	0.09	0.16	0.30	0.28	0.51**	−0.45*	−0.36*	–		
10 Share of rural population	0.71***	0.59**	0.55**	0.44*	0.48**	0.75***	−0.92***	−0.82***	0.36*	–	
11 Share of college students	−0.62***	−0.65***	−0.46**	−0.44*	−0.28	−0.62***	0.82***	0.76***	−0.21	−0.89***	–
M	0.15	0.11	0.07	0.39	0.11	0.04	4887.42	0.24	557.50	0.68	0.01
SD	0.08	0.05	0.05	0.02	0.01	0.00	2466.46	0.28	598.26	0.17	0.05

^†^The emotional variable has been multiplied by 100, ***p < 0.001, **p < 0.01, *p < 0.05.

To examine whether online disgust could predict nationalism at the regional level after controlling other confounding variables, we establish the regression models by using the ordinary-least-squares method. The results of the regression analysis are presented in Table 2.

**TABLE 2:** Nationalism and online disgust across 31 provinces in China, OLS Estimates.

	**Model 1 Beta**	**Model 2 Beta**	**Model 3 Beta**
Economic openness	−0.51*	−0.54*	−0.50*
Geographical closeness	0.22	0.26	0.33*
GDP per capita	−0.20	−0.12	−0.15
Share of rural population	0.04	0.06	0.06
Share of college students	−0.01	0.17	0.10
Online disgust		0.31*	0.40*
Online anger			−0.00
Online happiness			−0.09
Online fear			0.14
Online sadness			−0.21
Δ*R*^2^	0.65***	0.05*	0.03

***p < 0.001, **p < 0.01, *p < 0.05.

*OLS: ordinary-least-squares; Beta: standardized regression coefficients.*

Results show that, after controlling the confounding variables, disgust also predicted nationalism at the province level (β = 0.31, *t* = 2.06, *p* = 0.049, △*R*^2^ = 0.05) although *R*^2^ changed approximately 5% after adding disgust in *Model* 2. Given that there were only 31 cases (provinces) in the models, the result was considered acceptable.

#### Discriminant Validity Test

Previous studies find that both disgust and anger in basic emotions are associated with negative outgroup evaluations (Rozin et al., 1999). The correlation analysis showed that disgust, anger, and sadness are highly correlated (see Table 1). Therefore, we conducted the analysis of discriminative validity to determine whether disgust has a unique influence on nationalism. We used another four basic emotions—happiness, anger, fear, and sadness—to predict nationalism while controlling other variables used earlier. We obtained the standardized regression coefficients of four emotions ranging from -0.03 to 0.19 (*p*s > 0.05). Finally, we added all the emotions to the regression equation in *Model* 3 (see Table 2). Results show that, after controlling the confounding variables, merely online disgust significantly predicted nationalistic orientation at the regional level (β = 0.40, *t* = 2.11, *p* = 0.047). This verified our hypothesis. At the regional level, the association between disgust and nationalistic attitude remained valid even after controlling for variables related to nationalism.

## Discussion

### Disgust and Nationalism

We tested our hypothesis that disgust can predict nationalism at both individual and regional levels. At the individual level, we show that disgust can increase an individual’s nationalistic orientation by a laboratory experiment. This method ensured the internal validity of the study and avoided the effects of omitted-variable bias. Hence, we identified the causal relationship between disgust and nationalism. Previous studies have found that disgust has a deeper structure, such as core disgust, animal reminders, interpersonal disgust, moral disgust, sexual disgust, pathogen disgust, and so on (Rozin et al., 2000; Tybur et al., 2009). Our experimental materials induced pathogen disgust more than any other kind of disgust. Hence, the relationship between nationalism and the other kinds of disgust needs further verification as our research was more concerned with the disease avoidance response of disgust. However, from an evolutionary perspective, these domains of disgust may be unified by using the framework of the behavioral immune system. Schaller and Murray (2008) describe the behavioral immune system’s function as cognitive detection of pathogens, an emotional experience of fear and disgust, and a behavioral reaction. Because the behavioral immune system mechanisms underlying pathogen disgust—regardless of the cues that led to the disgust—could act as an input into attention and memory systems, disgust across stimulus types could have similar effects on cognition (Tybur et al., 2013; Schaller, 2014). Therefore, we believe that other kinds of disgust should arouse an individual’s nationalism to a greater or lesser degree.

Moreover, through big data research, we find that the relationship between disgust and nationalism still exist at the macro level. The results suggest a positive relationship between online disgust and nationalism at the individual and regional levels. The more disgust people of a given region expressed online, the stronger the nationalism of that region. We found this relationship still existed even after controlling variables used in previous studies. This shows that emotional state could explain variation in nationalism independent of socioeconomic variables at a regional level; indeed, with online disgust, economic openness, and geographical closeness, the model could explain more than 70% of the variation in nationalism at the regional level. Although online disgust may capture only part of the variance in nationalist attitude, considering the research results at the individual level, we still have confidence in the relationship between disgust and nationalism at the regional level. As for the socioeconomic variables, we find that economic openness had the highest explanatory power for nationalism, and this result was very stable. This result validated the previous findings of Lan and Li (2015). A region’s nationalism depends more on the region’s economic interests in its foreign market relative to the domestic market rather than on the area’s absolute economic level. In other words, the greater the share of international trade in a region’s economy, the lower the nationalism of the people in that region. Conversely, if a region’s economy is entirely self-sufficient, then nationalism is highest in that region. In China, reform and opening up began earliest in the coastal regions, which have the largest share of foreign trade. From Figure 2, we can see that nationalism is lower in the coastal areas and higher in the inland provinces.

Disgust can both be a chronical trait (disgust sensitivity) and a temporary state (Ekman et al., 1987; Haidt et al., 1994; Inbar et al., 2012). In the current study, participants who were chronically sensitive to disgust (Study 2) and who were temporarily induced to feel disgusted (vs. control, Study 1) were more likely to adopt nationalist ideology. In terms of the causal relationship between disgust and nationalism, Study 1 demonstrates through an emotion induction experiment that manipulating an individual’s disgust can influence an individual’s nationalism. Participants temporally induced disgust (vs. control) increased the adoption of nationalism. Study 1 proved the causal relationship between disgust and nationalism. As for Study 2, we analyzed covariation in online disgust and nationalism at the province level. The results show that, after controlling other variables, disgust also predicted nationalism at the province level.

It should be noted that the results of Study 2, correlational ones, do not provide direct causal evidence for the relation between disgust and nationalism. However, considering that Study 1 demonstrates the causal association between disgust and nationalism, the results of Study 2 do provide insights into the causality between the two foci variables, especially at the macro level. In addition, previous correlational studies of disgust have often considered disgust as a predictor variable of social perceptions and attitudes (Inbar et al., 2009a; Brenner and Inbar, 2015). Further, the cross-sectional studies at the regional level commonly identified the theoretical independent variables as predictor ones (e.g., Brethel-Haurwitz and Marsh, 2014; Obschonka et al., 2016). Therefore, we describe disgust as a predictor variable of nationalism in Study 2 to keep internal consistency with the theoretical hypothesis and the results in Study 1.

The concept of nationalism is more complex than ethnocentrism and xenophobia as it includes hostility toward foreign groups and the need for national power and dominance. In line with terror management theory, disgust increases an individual’s death-related thoughts, leading to power-seeking (Belmi and Pfeffer, 2016). Taken together with other researchers’ findings (Park et al., 2003; Navarrete et al., 2007; Fincher and Thornhill, 2012; Terrizzi et al., 2012), we find that disgust plays an important role in dealing with outgroups, such as in ethnocentrism and xenophobia. The current study advances research in the field of disgust and nationalism.

We did not find that anger could predict nationalism at the regional level, which is not consistent with previous research that suggests anger increases ethnocentrism (Banks, 2016). Although ethnocentrism and nationalism are not precisely the same, we consider the main reason why anger did not predict nationalism: anger has less influence on nationalism, especially for less tension internationally, so the effect was not reflected at the macro-level. Although disgust, contempt, and anger are all emotions that condemn others, their functions have also been proven to be differentiated by researchers (Rozin et al., 1999). Our finding confirms the unique impact of disgust on nationalism.

The current study indicates that people who are exposed to disgusting stimuli have a higher level of nationalism. As an evolved disease-avoidance mechanism, disgust is related to pathogens (Faulkner et al., 2004). This indicates a close relationship between disease prevalence and nationalism. Recently, researchers believe that global nationalism would rise in times of the COVID-19 pandemic (Bieber, 2020). Our study provides a potential theoretical basis and research evidence for this issue.

### Limitations and Future Directions

Several limitations of this study should be acknowledged. First, in terms of research methodology, the current study expanded both studies’ sample sizes. In Study 1, we recruited more than 800 participants for the experimental study. In Study 2, we analyzed the relationship between disgust and nationalism through 167 million micro-blogging posts and more than 150,000 surveys. Social media data has advanced the psychological research so far, but the sample representativeness is still an issue not to be ignored. The samples of Studies 1 and 2 consist mainly of university students and internet users who were younger and more educated than the average Chinese citizens. Future research requires samples of more diverse backgrounds, such as a full range of ages and educations. Besides this, we speculate that the relationship between nationalism and disgust may exist in multiple cultures. As with disgust and conservatism, people in different cultures share similar psychological mechanisms (Inbar et al., 2009a; Wang et al., 2019). However, considering the cultural uniqueness of China, more research in other cultures is needed.

Second, in terms of theoretical depth, the current research theoretically analyzed the relationship between disgust and nationalism and examined this relationship at both individual and regional levels. However, we did not explore the underlying mechanism between disgust and nationalism. The study of the underlying psychological process can further reveal the relation between disgust and nationalism. In addition, it is also of great significance to explore which factors can moderate the effect of disgust on nationalism.

Last but not least, in terms of practical application, nationalism has been on the global rise in recent years and plays an essential role in many movements (Gusterson, 2017). Our study provides a new theoretical basis for understanding and predicting the issues of nationalism. Under the global pandemic of COVID-19, disgust serving as a disease-avoidance mechanism may lead to a higher level of nationalism. It remains to be further explored by researchers in the future. Besides this, there are many forms of nationalism, and our research only focuses on abstract nationalist attitudes. Therefore, it is of great value to analyze the relationship between disgust and specific nationalist activities (e.g., anti-globalization, anti-immigration) in the future. This research would further provide practical advice for addressing real-world problems.

## Conclusion

Overall, these studies provided robust evidence to confirm a relationship between disgust and nationalism at both individual and regional levels. Significantly, the current study utilizes big data from the social network websites to analyze the relationship between online disgust expression and nationalism at the regional level, linking the micro psychological mechanism and macro decision-making judgment, which researchers will further explore in the future.

## Data Availability Statement

The experimental datasets and Sina Weibo datasets for this study are available from the corresponding author, upon request. The Chinese Political Compass datasets belong to its website, and can be found and downloaded from http://www.zuobiao.me.

## Ethics Statement

The studies involving human participants were reviewed and approved by the Research Ethics Review Committee of Department of Social Psychology, Nankai University. The patients/participants provided their written informed consent to participate in this study.

## Author Contributions

All authors contributed to the writing of the manuscript and approved the final manuscript to be published. SG and HC conducted data analysis and wrote the first draft of the manuscript. KL revised the manuscript. WQ crawled and cleaned *Weibo*’s big data.

## Conflict of Interest

The authors declare that the research was conducted in the absence of any commercial or financial relationships that could be construed as a potential conflict of interest.

## Footnotes

1 According to the Weibo API Blog, *Weibo* officially upgraded the API access permission in 2014. It resulted in difficulty in data collecting via the *Weibo* API and an inability to match the 2011–2013 data. Thus, we did not analyze *Weibo*’s data of 2014 and after.

2 www.zuobiao.me

3 www.stats.gov.cn

## References

[B1] Angraini Y. Toharudin T. Folmer H. Oud J. H. L. (2014). The relationships between individualism, nationalism, ethnocentrism, and authoritarianism in flanders: a continuous time-structural equation modeling approach. *Multivariate Behav. Res.* 49 41–53. 10.1080/00273171.2013.836621 26745672

[B2] Banks A. J. (2016). Are group cues necessary? How anger makes ethnocentrism among whites a stronger predictor of racial and immigration policy opinions. *Polit. Behav*. 38 635–657. 10.1007/s11109-016-9330-3

[B3] Belmi P. Pfeffer J. (2016). Power and death: mortality salience increases power seeking while feeling powerful reduces death anxiety. *J. Appl. Psychol.* 101 702–720. 10.1037/apl0000076 26867106

[B5] Bieber F. (2020). Global Nationalism in Times of the COVID-19 Pandemic. *Natl. Pap*. 2020 1–13. 10.1017/nps.2020.35

[B6] Bizumic B. Duckitt J. (2012). What is and is not ethnocentrism? A conceptual analysis and political implications. *Polit. Psychol.* 33 887–909. 10.1111/j.1467-9221.2012.00907.x

[B7] Bollen J. Mao H. Zeng X. (2011). Twitter mood predicts the stock market. *J. Comput. Sci.* 2 1–8. 10.1016/j.jocs.2010.12.007

[B100] Brenner C. J. Inbar Y. (2015). Disgust sensitivity predicts political ideology and policy attitudes in the Netherlands. *Eur. J. Soc. Psychol.* 45, 27–38. 10.1002/ejsp.2072

[B8] Brethel-Haurwitz K. M. Marsh A. A. (2014). Geographical differences in subjective well-being predict extraordinary altruism. *Psychol. Sci.* 25 762–771. 10.1037/e514472015-782 24477966

[B9] Charash M. McKay D. (2002). Attention bias for disgust. *J. Anxiety Disord*. 16 529–541. 10.1016/S0887-6185(02)00171-8 12396210

[B10] Cheon B. K. Christopoulos G. I. Hong Y. Y. (2016). Disgust associated with culture mixing: why and who? *J. Cross Cult. Psychol.* 47 1268–1285. 10.1177/0022022116667845

[B11] Cox C. R. Goldenberg J. L. Pyszczynski T. Weise D. (2007). Disgust, creatureliness and the accessibility of death-related thoughts. *Eur. J. Soc. Psychol.* 37 494–507. 10.1002/ejsp.370

[B12] Dasgupta N. Desteno D. Williams L. A. Hunsinger M. (2009). Fanning the flames of prejudice: the influence of specific incidental emotions on implicit prejudice. *Emotion* 9 585–591. 10.1037/a0015961 19653784

[B13] Dong Y. Chen H. Tang X. Qian W. Zhou A. (2015). “Prediction of social mood on Chinese societal risk perception” in *2015 International Conference on Behavioral, Economic and Socio-Cultural Computing (BESC).* (Piscataway: IEEE). 10.1109/besc.2015.7365966

[B14] Dong Y. H. Chen H. Lai K. X. Yue G. A. (2015a). Weibo Social Moods Measurement and Validation. *J. Psychol. Sci.* 38 1141–1146. 10.16719/j.cnki.1671-6981.2015.05.034

[B15] Dong Y. H. Chen H. Qian W. N. Zhou A. Y. (2015b). Micro-blog social moods and Chinese stock market: the influence of emotional valence and arousal on shanghai composite index volume. *Int. J. Embed. Syst.* 7:148. 10.1504/IJES.2015.069987

[B16] Eichstaedt J. C. Schwartz H. A. Kern M. L. Park G. Labarthe D. R. Merchant R. M. (2015). Psychological language on twitter predicts county-level heart disease mortality. *Psychol. Sci.* 26 159–169. 10.1177/0956797614557867 25605707 PMC4433545

[B17] Ekman P. Friesen W. V. O’Sullivan M. Chan A. Diacoyanni-Tarlatzis I. Heider K. (1987). Universals and cultural differences in the judgments of facial expressions of emotion. *J. Pers. Soc. Psychol.* 53 712–717. 10.1037/0022-3514.53.4.712 3681648

[B18] Faulkner J. Schaller M. Park J. H. Duncan L. A. (2004). Evolved disease-avoidance mechanisms and contemporary xenophobic attitudes. *Group Process. Intergroup Relat.* 7 333–353. 10.1177/1368430204046142

[B19] Fincher C. L. Thornhill R. (2012). Parasite-stress promotes in-group assortative sociality: the cases of strong family ties and heightened religiosity. *Behav. Brain Sci.* 35 1–59. 10.1017/S0140525X11000021 22289223

[B20] Freeden M. (1998). Is nationalism a distinct ideology? *Polit. Stud.* 46 748–765. 10.1111/1467-9248.00165

[B21] Goode J. P. Stroup D. R. Gaufman E. (2020). Everyday Nationalism in Unsettled Times: in Search of Normality during Pandemic. *Natl. Pap.* 1–25. 10.1017/nps.2020.40 [online ahead of print]

[B22] Gusterson H. (2017). From Brexit to Trump: anthropology and the rise of nationalist populism. *Am. Ethnol.* 44 209–214. 10.1111/amet.12469

[B23] Haidt J. McCauley C. Rozin P. (1994). Individual differences in sensitivity to disgust: a scale sampling seven domains of disgust elicitors. *Pers. Individ. Dif.* 16 701–713. 10.1016/0191-8869(94)90212-7

[B24] Hodson G. Choma B. L. Boisvert J. Hafer C. L. MacInnis C. C. Costello K. (2013). The role of intergroup disgust in predicting negative outgroup evaluations. *J. Exp. Soc. Psychol.* 49 195–205. 10.1016/j.jesp.2012.11.002

[B25] Hodson G. Costello K. (2007). Interpersonal disgust, ideological orientations, and dehumanization as predictors of intergroup attitudes. *Psychol. Sci.* 18 691–698. 10.1111/j.1467-9280.2007.01962.x 17680940

[B26] Inbar Y. Pizarro D. A. Bloom P. (2009a). Conservatives are more easily disgusted than liberals. *Cogn. Emot.* 23 714–725. 10.1080/02699930802110007

[B27] Inbar Y. Pizarro D. A. Iyer R. Haidt J. (2012). Disgust sensitivity, political conservatism, and voting. *Soc. Psychol. Personal. Sci.* 3 537–544. 10.1177/1948550611429024

[B28] Inbar Y. Pizarro D. A. Knobe J. Bloom P. (2009b). Disgust sensitivity predicts intuitive disapproval of gays. *Emotion* 9 435–439. 10.1037/a0015960 19485621

[B29] Kosinski M. Matz S. C. Gosling S. D. Popov V. Stillwell D. (2015). Facebook as a research tool for the social sciences: opportunities, challenges, ethical considerations, and practical guidelines. *Am. Psychol.* 70 543–556. 10.1037/a0039210 26348336

[B30] Kosterman R. Feshbach S. (1989). Toward a measure of patriotic and nationalistic attitudes. *Polit. Psychol.* 10 257–274. 10.2307/3791647

[B31] Lai K. X. Chen H. Yue G. A. Dong Y. X. (2014). Can mood predict stock Market? *Adv. Psychol. Sci.* 22 1770–1780. 10.3724/SP.J.1042.2014.01770

[B32] Lan X. Li B. G. (2015). The economics of nationalism. *Am. Econ. J. Econ. Policy* 7 294–325. 10.1257/pol.20130020

[B33] Luo Y. Shen W. Zhang Y. Feng T. Y. Huang H. Li H. (2013). Core disgust and moral disgust are related to distinct spatiotemporal patterns of neural processing: an event-related potential study. *Biol. Psychol.* 94 242–248. 10.1016/j.biopsycho.2013.06.005 23816951

[B34] Mitchell L. Frank M. R. Harris K. D. Dodds P. S. Danforth C. M. (2013). The geography of happiness: connecting twitter sentiment and expression, demographics, and objective characteristics of place. *PLoS One* 8:e64417. 10.1371/journal.pone.0064417 23734200 PMC3667195

[B35] Mummendey A. Klink A. Brown R. (2001). A rejoinder to our critics and some of their misapprehensions. *Br. J. Soc. Psychol.* 40, 187–191. 10.1348/014466601164803 11446226

[B36] Mummendey A. Klink A. Brown R. (2011). Nationalism and patriotism: national identification and out-group rejection. *Br. J. Soc. Psychol.* 40 159–172. 10.1348/014466601164740 11446222

[B37] Navarrete C. D. Fessler D. M. Eng S. J. (2007). Elevated ethnocentrism in the first trimester of pregnancy. *Evol. Hum. Behav.* 28 60–65. 10.1016/j.evolhumbehav.2006.06.002

[B38] Navarrete C. D. Fessler D. M. T. (2006). Disease avoidance and ethnocentrism: the effects of disease vulnerability and disgust sensitivity on intergroup attitudes. *Evol. Hum. Behav.* 27 270–282. 10.1016/j.evolhumbehav.2005.12.001

[B39] Nielsen F. Å (2011). A new ANEW: evaluation of a word list for sentiment analysis in microblogs. *ArXiv* 1103 93–98.

[B40] Obschonka M. Stuetzer M. Audretsch D. B. Rentfrow P. J. Potter J. Gosling S. D. (2016). Macropsychological factors predict regional economic resilience during a major economic crisis. *Soc. Psychol. Pers. Sci.* 7 95–104. 10.1177/1948550615608402

[B41] O’Connor B. Balasubramanyan R. Routledge B. R. Smith N. A. (2010). “From Tweets to Polls: linking Text Sentiment to Public Opinion Time Series” in *Proceedings of the Fourth International Conference on Weblogs and Social Media, ICWSM 2010*, (California: AAAI Press) 1–2.

[B42] Oishi S. (2014). Socioecological psychology. *Annu. Rev. Psychol.* 65 581–609. 10.1146/annurev-psych-030413-15215 23987114

[B43] Park J. H. Faulkner J. Schaller M. (2003). Evolved disease-avoidance processes and contemporary anti-social behavior: prejudicial attitudes and avoidance of people with physical disabilities. *J. Nonverbal. Behav.* 27 65–87. 10.1023/A:102391040

[B44] Rentfrow P. J. Gosling S. D. Potter J. (2008). A theory of the emergence, persistence, and expression of geographic variation in psychological characteristics. *Perspect. Psychol. Sci.* 3 339–369. 10.1111/j.1745-6924.2008.00084.x 26158954

[B45] Rentfrow P. J. Jost J. T. Gosling S. D. Potter J. (2009). “Statewide Differences in Personality Predict Voting Patterns in 1996–2004 U.S. Presidential Elections” in *Social and Psychological Bases of Ideology and System Justification*, eds Jost J. T. Kay A. C. Thorisdottir H. (Oxford : Oxford University Press), 314–348. 10.1093/acprof:oso/9780195320916.003.013

[B46] Rothschild L. (2015). Nationalism and the body politic: psychoanalysis and the rise of ethnocentrism and xenophobia. *Psychoanal. Cult. Soc.* 20 421–423. 10.1057/pcs.2015.33

[B47] Rozin P. Fallon A. E. (1987). A perspective on disgust. *Psychol Rev.* 94 23–41. 10.1037/0033-295X.94.1.23 3823304

[B48] Rozin P. Haidt J. McCauley C. (2000). “Disgust,” in *Handbook of Emotions* (2nd ed), eds Lewis M. Haviland Jones S. M. (New York: Guilford Press), 637–653.

[B49] Rozin P. Haidt J. McCauley C. R. (2008). “Disgust,” in *Handbook of Emotions* (2nd ed), eds Lewis M. Haviland J. (New York, NY: Guilford Press), 757–776.

[B50] Rozin P. Lowery L. Imada S. Haidt J. (1999). The CAD triad hypothesis: a mapping between three moral emotions (contempt, anger, disgust) and three moral codes (community, autonomy, divinity). *J. Pers. Soc. Psychol.* 76 574–586. 10.1037/0022-3514.76.4.574 10234846

[B51] Saif H. He Y. Alani H. (2012). “Semantic sentiment analysis of twitter,” in *The Semant Web ISWC 2012*, eds Cudré-Mauroux P. Heflin J. Sirin E. Tudorache T. (Berlin: Springer), 508–524.

[B52] Schaller M. (2014). When and how disgust is and is not implicated in the behavioral immune system. *Evol. Behav. Sci.* 8 251–256. 10.1037/ebs0000019

[B53] Schaller M. Murray D. R. (2008). Pathogens, personality, and culture: disease prevalence predicts worldwide variability in sociosexuality, extraversion, and openness to experience. *J. Pers. Soc. Psychol.* 95 212–221. 10.1037/0022-3514.95.1.212 18605861

[B54] Schnall S. Haidt J. Clore G. L. Jordan A. H. (2008). Disgust as embodied moral judgment. *Pers. Soc. Psychol. Bull.* 34 1096–1109. 10.1177/0146167208317771 18505801 PMC2562923

[B55] Suedfeld M. Schaller P. (2002). “Authoritarianism and the holocaust: Some cognitive and affective implications,” in *Understanding GenocideThe Social Psychology of the Holocaust*, eds Newman L. S. Erber R. (Oxford: Oxford University Press), 68–90. 10.1093/acprof:oso/9780195133622.003.0004

[B56] Terrizzi J. A. Jr. Shook N. J. Ventis W. L. (2010). Disgust: a predictor of social conservatism and prejudicial attitudes toward homosexuals. *Pers. Individ. Dif*. 49 587–592. 10.1016/j.paid.2010.05.024

[B57] Terrizzi J. A. Jr. Shook N. J. Ventis W. L. (2012). Religious conservatism: an evolutionarily evoked disease-avoidance strategy. *Religion Brain Behav.* 2 105–120. 10.1080/2153599X.2012.695514

[B58] Tybur J. M. Lieberman D. Griskevicius V. (2009). Microbes, mating, and morality: individual differences in three functional domains of disgust. *J. Pers. Soc. Psychol*. 97 103–122. 10.1037/a0015474 19586243

[B59] Tybur J. M. Lieberman D. Kurzban R. DeScioli P. (2013). Disgust: evolved function and structure. *Psychol. Rev.* 120 65–84. 10.1037/a0030778 23205888

[B60] Wang R. Yang Q. Huang P. Sai L. (2019). The association between disgust sensitivity and negative attitudes toward homosexuality: the mediating role of moral foundations. *Front. Psychol.* 10:1229. 10.3389/fpsyg.2019.01229 31244709 PMC6562335

[B61] Weibo Data Center. (2014). *2014 Weibo user development report.* URL: http://data.weibo.com/report/reportDetail?id=215

[B62] Wu A. X. (2014). Ideological polarization over a China-as-superpower mindset: an exploratory charting of belief systems among Chinese Internet users, 2008-2011. *Int. J. Commun* 8 2243–2272.

[B63] Wu M. S. Zhou C. Chen H. Cai H. J. Sundararajan L. (2018). Cultural value mismatch in urbanizing China: a large-scale analysis of collectivism and happiness among social media and individuals. *Int. J. Psychol.* 53 54–63. 10.1002/ijop.12523 30239987

[B64] Żelaźniewicz A. Pawłowski B. (2015). Disgust in pregnancy and fetus sex—Longitudinal study. *Physiol. Behav.* 139 177–181. 10.1016/j.physbeh.2014.11.032 25449396

